# Nonsmall Cell Lung Carcinoma with Giant Cell Features Expressing Programmed Death-Ligand 1: A Report of a Patient Successfully Treated with Pembrolizumab

**DOI:** 10.1155/2018/5863015

**Published:** 2018-03-15

**Authors:** Shingo Nakayama, Mamoru Sasaki, Shojiroh Morinaga, Naoto Minematsu

**Affiliations:** ^1^Department of Medicine, Hino Municipal Hospital, 4-3-1 Tamadaira, Hino-shi, Tokyo 191-0062, Japan; ^2^Department of Diagnostic Pathology, Hino Municipal Hospital, 4-3-1 Tamadaira, Hino-shi, Tokyo 191-0062, Japan

## Abstract

Giant cell carcinoma, a rare variant of nonsmall cell lung carcinoma (NSCLC), is characterized by aggressive progression and poor response to conventional chemotherapy. This report is the first to describe a patient with NSCLC and giant cell features who was successfully treated with pembrolizumab, an antibody targeting programmed death-1 (PD-1). A 69-year-old woman was diagnosed with NSCLC with multiple brain metastases. Histological evaluation of lung biopsy specimens revealed proliferation of pleomorphic giant tumor cells with poor cohesiveness, findings consistent with giant cell carcinoma. Immunostaining showed that a high proportion of the tumor cells were positive for expression of programmed death-ligand 1 (PD-L1). The patient received stereotactic radiotherapy for the brain metastases, followed by administration of pembrolizumab. Treatment with pembrolizumab resulted in the rapid regression of the primary lung nodule, with the progression-free period maintained for at least four treatment cycles. Immunotherapy targeting PD-1/PD-L1 may be an option for patients with PD-L1-positive NSCLC with giant cell features.

## 1. Introduction

Giant cell carcinoma is a rare variant of nonsmall cell lung carcinoma (NSCLC) that is clinically characterized by aggressive progression and poor response to antitumor chemotherapy. This report describes a patient with NSCLC and giant cell features positive for high tumor expression of programmed death-ligand 1 (PD-L1). Administration of pembrolizumab, an anti-programmed death-1 (PD-1) antibody, resulted in rapid tumor regression.

## 2. Case Presentation

**Figure 1:**
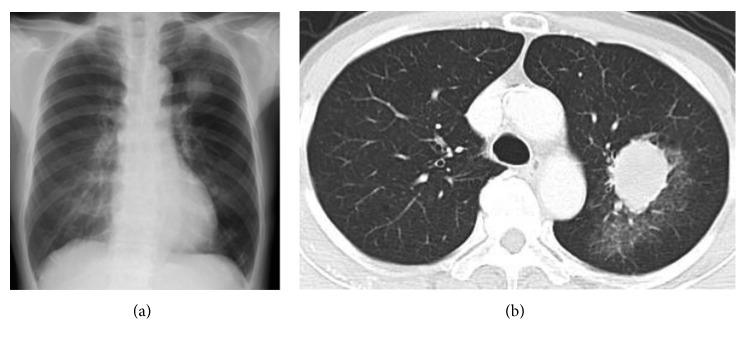
Chest X-ray showing an apparent nodule in the left upper lung field (a). Contrast-enhanced computed tomography of the chest, showing a large nodule with surrounding ground-glass opacity in the left upper lobe of the lung (b).

A 69-year-old woman was referred to our hospital because of prolonged bloody sputum and a lung nodule on chest X-ray. She was a never-smoker and had no previous relevant medical history. Her chest X-ray suggested a nodule in the left upper lung field (Figure 1(a)), and a contrast-enhanced computed tomography (CT) scan revealed a large nodule (41 × 33 mm in size) with surrounding ground-glass opacity in the left upper lobe of the lung (Figure 1(b)). There was no evidence of intrathoracic lymph node enlargement or extrathoracic metastasis, but gadolinium-enhanced magnetic resonance imaging (MRI) of the head revealed three nodules in both cerebral hemispheres (13 mm in maximum size), suggesting multiple brain metastases. A 2-[^18^F]-fluoro-2-deoxy-D-glucose (FDG) positron emission tomography (PET) scan showed a high maximum standardized uptake value (SUV) of 28.4 in the lung nodule and marginal uptake in the small lymph nodes of the mediastinum (maximum SUV 3.49). The ground-glass opacity surrounding the primary tumor was focally positive for FDG accumulation, suggesting a mixture of carcinomatous and noncarcinomatous components.

**Figure 2:**
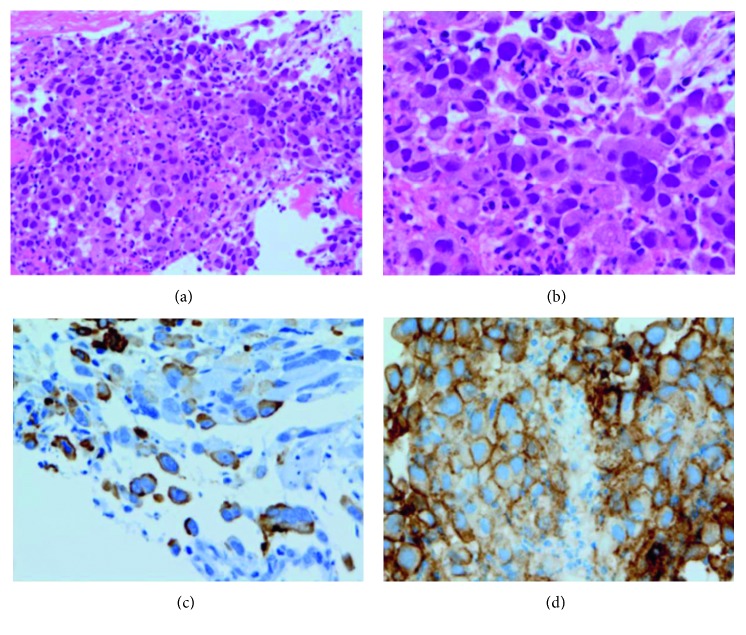
Histological examination of a lung biopsy specimen at low (a) and high (b) magnifications, showing pleomorphic giant tumor cells with poor cohesiveness. No apparent differentiation toward adenocarcinoma, squamous cell carcinoma, or neuroendocrine carcinoma was seen. Immunostaining showed that the tumor cells were positive for expression of CAM5.2 (c) and PD-L1 (d).

The patient was suspected of having a stage IVB primary lung cancer, and a diagnostic bronchoscopy was scheduled. Tumor specimens were obtained from the lung nodule by transbronchial biopsy with the assistance of sheath-guided endobronchial ultrasonography. Microscopically, the biopsy specimens were almost entirely composed of pleomorphic giant tumor cells with poor cohesiveness, with some of these cells being multinucleated (Figures 2(a) and 2(b)). There was no evidence of adenocarcinoma, squamous cell carcinoma, and neuroendocrine carcinoma components. Immunostaining showed that the biopsy specimens were positive for expression of cytokeratin CAM5.2 (Figure 2(c)), but negative for all other markers examined, including thyroid transcription factor 1, napsin A, p40, chromogranin, synaptophysin, CD56, and human chorionic gonadotropin *β* subunit. Taken together, the pathological features of this tumor were consistent with a giant cell carcinoma of the lung. Based on 2015 World Health Organization (WHO) [1] and 2011 International Association for the Study of Lung Cancer/American Thoracic Society/European Respiratory Society (IASLC/ATS/ERS) [2] classifications, the patient was pathologically diagnosed with NSCLC with giant cell features. The tumor was negative for somatic alterations of the *epidermal growth factor receptor* (*EGFR*) and *anaplastic large kinase* (*ALK*) genes. However, PD-L1 expression was positive in a high proportion of tumor cells (tumor proportion score (TPS), 75%; Figure 2(d)) by immunostaining using a companion diagnostic kit, PD-L1 IHC 22C3 pharmDx Dako (Agilent, Santa Clara, CA, USA).

**Figure 3:**
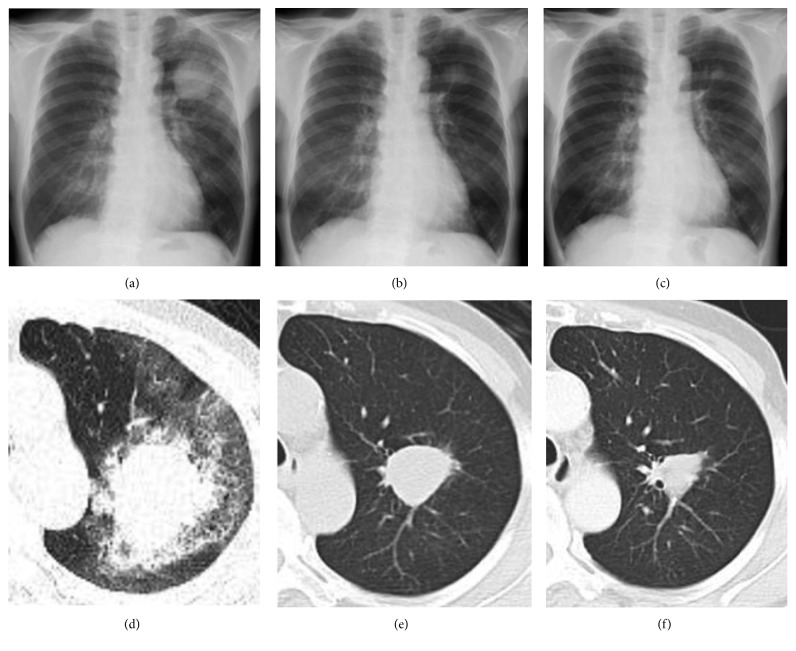
Diameters of the primary tumor before starting treatment with pembrolizumab (a and d), and after two (b and e) and four (c and f) cycles of treatment.

While the brain metastases were asymptomatic, the patient received stereotactic radiotherapy to avoid the risk of developing neurological symptoms. Two weeks after brain radiotherapy and five weeks from the first visit, she was admitted to our hospital to initiate antitumor treatment. The chest X-ray on admission (Figure 3(a)), as well as the CT scan three weeks before the admission (Figure 3(d)), showed rapid enlargement of the primary tumor, compared with assessments at the initial visit. Because a high proportion of tumor cells were positive for PD-L1, she was eligible for first-line treatment with pembrolizumab according to the Japanese Clinical Practice Guideline [3]. The patient consented to treatment and was administered intravenous pembrolizumab (200 mg on day 1, every three weeks), starting the day after admission. The primary lung nodule showed marked regression, and the peripheral ground-glass opacity dimmed after one week. The patient was discharged from the hospital and continued pembrolizumab treatment in the outpatient department. The lung nodule measured 32 × 23 mm after two pembrolizumab cycles (6 weeks) (Figures 3(b) and 3(e)) and 24 × 16 mm after four cycles (12 weeks) (Figures 3(c) and 3(f)). Moreover, the brain metastases disappeared after four cycles. The only adverse effect was grade 2 renal dysfunction (Common Terminology Criteria for Adverse Events v4.0, https://www.evs.nci.nih.gov/).

## 3. Discussion

This report describes a patient with NSCLC with giant cell features, in which a high proportion of cells were positive for PD-L1 expression. Immunotherapy targeting PD-1 had a rapid and excellent antitumor effect. To our knowledge, this is the first report describing the treatment of this rare carcinoma with anti-PD-1 antibodies.

Giant cell carcinoma is a rare pathological subtype of lung cancers, accounting for less than 1% of all lung cancers in an epidemiological study and 2-3% in a surgical series [4]. This tumor has been categorized as a sarcomatoid carcinoma, along with other subtypes, including pleomorphic carcinoma, spindle cell carcinoma, carcinosarcoma, and pulmonary blastoma. WHO [1] and IASLC/ATS/ERS [2] guidelines include the pathological terminology and criteria that should be applied in small biopsy specimens, as most patients with inoperable tumors are diagnosed by pathologic examination of small samples, and many of these lung tumors are pathologically heterogeneous. Tumors are diagnosed as giant cell carcinomas if more than 10% of the cells in surgical specimens are giant cells [1, 2]. However, biopsy specimens containing a component of giant cell carcinoma are diagnosed as NSCLC (or, e.g., adenocarcinoma or squamous cell carcinoma) with giant cell features, regardless of the proportion of the latter. Thus, despite the biopsy specimens from our patient consisting almost entirely of pleomorphic giant tumor cells without other components, she was diagnosed with NSCLC with giant cell features.

Clinically, giant cell carcinomas are characterized by aggressive growth and poor response to antitumor chemotherapy, resulting in poor patient prognosis [5, 6]. Indeed, the tumor in our patient grew dramatically during diagnostic evaluation for a few weeks. A personalized treatment strategy may improve prognosis in a subset of NSCLC patients with somatic alternations in driver genes, including *EGFR* and *ALK*. Although limited information is currently available about somatic mutations in the driver genes of giant cell carcinoma [7], assessments of a small number of subjects found that the EGFR tyrosine kinase inhibitors were effective for giant cell carcinomas with *EGFR* sensitive mutations [6]. Pembrolizumab was recently shown to have a greater antitumor effect than platinum-containing combination chemotherapy against NSCLC with high expression of PD-L1 (TPS ≥ 50%) [8]. Little is known about PD-L1 protein expression in lung giant cell carcinomas, although one report showed positive PD-L1 expression in 6 of 10 tumors [9]. Immunohistochemical assays showed that PD-L1 was expressed by 37 (90.2%) of 41 pleomorphic carcinomas of the lung, another type of sarcomatoid carcinoma, with PD-L1 expression being higher in the sarcomatoid than in the carcinomatous area [10]. Additional studies are required to determine whether sarcomatoid carcinomas expressing high levels of PD-L1 can be successfully treated by immunotherapy targeting PD-1/PD-L1.

In summary, this report is, to our knowledge, the first to describe a patient with a PD-L1-positive giant cell carcinoma of the lung who was successfully treated with the anti-PD-L1 antibody, pembrolizumab.
